# The 9th Brazilian Congress of Social and Human Sciences in Health of Abrasco: betting on the emancipatory power of plural and inclusive exchanges

**DOI:** 10.1590/0102/311XEN203123

**Published:** 2023-12-11

**Authors:** Mônica de Oliveira Nunes de Torrenté, Suely Deslandes, Marta Inez Machado Verdi, Rui Massato Harayama, Luís Henrique da Costa Leão, Keila Silene de Brito e Silva

**Affiliations:** 1 Instituto de Saúde Coletiva, Universidade Federal da Bahia, Salvador, Brasil.; 2 Instituto Nacional de Saúde da Mulher, da Criança e do Adolescente Fernandes Figueira, Fundação Oswaldo Cruz, Rio de Janeiro, Brasil.; 3 Universidade Federal de Santa Catarina, Florianópolis, Brasil.; 4 Universidade Federal do Oeste do Pará, Santarém, Brasil.; 5 Instituto de Saúde Coletiva, Universidade Federal de Mato Grosso, Cuiabá, Brasil.; 6 Centro Acadêmico de Vitória, Universidade Federal de Pernambuco, Vitória de Santo Antão, Brasil.

Another page in the history of Brazilian Public Health Association (Abrasco) was written from October 30 to November 3, 2023, during the 9th Brazilian Congress of Social and Human Sciences in Health ^1^ at the Federal University of Pernambuco. With the theme *Emancipation and Health: Decoloniality, Reparation, and Critical Reconstruction*, the event proposed a dialogue with previous editions and absorbed the discussions in the area, expressed in the Master Plan of the Commission of Social and Human Sciences in Health developed from 2020 to 2022. As it took place in a post-pandemic context, where democratic institutions were being re-established and public policy investment cycles strengthened, the debates focused on diversity, the decolonial purpose, and the demand for social reparation as guiding principles for both the production of public actions and the production and sharing of knowledge.

The dialogues and debates revolved around three major debates and 34 panel discussions on topics such as *Decoloniality of Can-be-know-do: Challenges and Contributions of Social and Human Sciences in Health*; *Repairing Historical Injustices in the Field of Health: Ethos, Knowledge, Politics, and Social Action*; and *Challenges for the Critical Reconstruction of Brazil: the Place and Contributions of Social and Human Sciences in Health*. Three panels were also held, guided by the preparatory discussions of the Congress, which sought to address central themes in the area, such as *The Distribution of Capital and the Question of Visibility in the Field of Public Health*; *The Critical Vanguard of Human Sciences in Health and their Potential in the Field of Public Health*; and *Training in (Public) Health in the Experience of Social and Human Sciences in Health*.

The significant participation of the Social and Human Sciences in Health community was evident and powerful, with almost 200 organizers and more than 2,300 participants at the Congress, 2,353 articles submitted and 1,770 presented in the 35 thematic meetings. We innovated methodologically by including representatives of the social movements on the scientific committee, who designed the activities with us and evaluated the articles submitted, and by holding meetings between generations of researchers and students (intergenerational coffees) and exchanges among researchers, students, and representatives of the social movements (inter-knowledge snacks).

The Congress brought many reflections on the role of Social and Human Sciences in Health in the paradigmatic renewal movements in the field, on the protagonism and diversity of epistemic subjects in a turn that affirms the plurality of rights, identities, and struggles. It also points to the urgent need to review parameters for the inclusion ^2^, visibility, and sustainability of our subfield in undergraduate and graduate programs. Moreover, the importance and role of the knowledge and practices of Social and Human Sciences in Health for the criticism, formulation, and reformulation of public policies based on and committed to democracy, human rights, and the defense of life were established. Therefore, the Congress took a stand, both in its opening speech and in the approval of a motion, for the defense of peace and against the brutal disregard for the humanitarian rules of international law, as is currently happening against the Palestinian people in the Gaza Strip.
